# The smoking cessation in pregnancy incentives trial (CPIT): study protocol for a phase III randomised controlled trial

**DOI:** 10.1186/s13063-019-4042-8

**Published:** 2020-02-14

**Authors:** Lesley Sinclair, Margaret McFadden, Helen Tilbrook, Alex Mitchell, Ada Keding, Judith Watson, Linda Bauld, Frank Kee, David Torgerson, Catherine Hewitt, Jennifer McKell, Pat Hoddinott, Fiona M. Harris, Isabelle Uny, Kathleen Boyd, Nicola McMeekin, Michael Ussher, David M. Tappin, for the CPIT III local research teams

**Affiliations:** 1 Usher Institute, University of Edinburgh, Teviot Place, Edinburgh, EH8 9AG UK; 2 Clinical R&D, Dykebar Hospital, Grahamstone Road, Paisley, PA2 7DE UK; 3 York Trials Unit, Department of Health Sciences, Faculty of Science, University of York, Alcuin Research Resource Centre, Heslington, York, YO10 5DD UK; 4 Centre of Excellence for Public Health, Queen’s University Belfast, University Road, Belfast, BT7 1NN UK; 5 Institute for Social Marketing, University of Stirling, Stirling, FK9 4LA UK; 6 NMAHP Research Unit, University of Stirling, Stirling, FK9 4LA UK; 7 Health Economics & Health Technology Assessment, Institute of Health & Wellbeing, 1 Lilybank Gardens, University of Glasgow, Glasgow, G12 8RZ UK; 8 Population Health Research Institute, St George’s, University of London, Cranmer Terrace, London, SW17 0RE UK; 9 Scottish Cot Death Trust, West Glasgow Ambulatory Care Hospital, Glasgow University, 5th Floor, Glasgow, G3 8SJ UK

**Keywords:** Intervention, Randomised controlled trial, Maternal and child health, Outcomes, Pregnancy, Prevention, Smoking cessation, Financial incentives

## Abstract

**Background:**

Eighty per cent of UK women have at least one baby, making pregnancy an opportunity to help women stop smoking before their health is irreparably compromised. Smoking cessation during pregnancy helps protect infants from miscarriage, still birth, low birth weight, asthma, attention deficit disorder and adult cardiovascular disease. UK national guidelines highlight lack of evidence for effectiveness of financial incentives to help pregnant smokers quit. This includes a research recommendation: within a UK context, are incentives an acceptable, effective and cost-effective way to help pregnant women who smoke to quit?

**Methods:**

The Cessation in Pregnancy Incentives Trial (CPIT) III is a pragmatic, 42-month, multi-centre, parallel-group, individually randomised controlled superiority trial of the effect on smoking status of adding to usual Stop Smoking Services (SSS) support, the offer of up to £400 of financial voucher incentives, compared with usual support alone, to quit smoking during pregnancy.

Participants (*n* = 940) are pregnant smokers (age > 16 years, < 24 weeks pregnant, English speaking), who consent via telephone to take part and are willing to be followed-up in late pregnancy and 6 months after birth.

The primary outcome is cotinine/anabasine-validated abstinence from smoking in late pregnancy. Secondary outcomes include engagement with SSS, quit rates at 4 weeks from agreed quit date and 6 months after birth, and birth weight. Outcomes will be analysed by intention to treat, and regression models will be used to compare treatment effects on outcomes. A meta-analysis will include data from the feasibility study in Glasgow. An economic evaluation will assess cost-effectiveness from a UK NHS perspective. Process evaluation using a case-study approach will identify opportunities to improve recruitment and learning for future implementation.

Research questions include: what is the therapeutic efficacy of incentives; are incentives cost-effective; and what are the potential facilitators and barriers to implementing incentives in different parts of the UK?

**Discussion:**

This phase III trial in Scotland, England and Northern Ireland follows a successful phase II trial in Glasgow, UK. The participating sites have diverse SSS that represent most cessation services in the UK and serve demographically varied populations. If found to be acceptable and cost-effective, this trial could demonstrate that financial incentives are effective and transferable to most UK SSS for pregnant women.

**Trial registration:**

Current Controlled Trials, ISRCTN15236311. Registered on 9 October 2017.

## Background and aims

Tobacco smoking is the leading preventable cause of death in the UK [1]. Individuals who give up by age 40 years (during childbearing years) avoid much of the morbidity and early mortality of continued smoking [2]; for example, lung cancer risk is reduced to two times that of never smokers compared with 16 times for lifelong smokers. Around 80% of UK women have at least one baby [3] so an effective intervention will eventually reach most women who smoke. Stopping smoking during pregnancy also reduces the likelihood of the children themselves becoming smokers [4], thus reducing future cancer risk.

Three hundred fifty UK still births each year [5] and a third of babies born small for gestational age are attributable to smoking during pregnancy. One-fifth of the 125,000 spontaneous miscarriages that occur each year in the UK [6], which cause 42,000 hospital admissions [7], are also associated with smoking during pregnancy. Compared with non-smokers, the relative risk of spontaneous miscarriage is 1.2 [8]. If causality was accepted, this 20% increase in risk would mean that 5000 spontaneous miscarriages and 2000 hospital admissions in the UK each year would be attributable to smoking during pregnancy.

Twenty per cent of sudden unexpected deaths in infancy and 9% of premature births are attributable to maternal smoking, as are 10% of admissions for bronchiolitis, one of the most common reasons why infants are admitted to hospital, and 7% of admissions for respiratory infection and asthma [9]. Perhaps, surprisingly, 12% of the rare but devastating occurrence of bacterial meningitis is attributable to maternal smoking [9], as are increases in attention deficit disorder [10] and learning difficulties [11] in children, adding substantial costs to health and social care services [12].

### Prevalence and available support for stopping smoking in pregnancy

UK pregnancy smoking rates remain high. One in four women smoke for part of their pregnancy and one in eight smoke throughout [13]. Stop Smoking Services (SSS) usually offer counselling plus free nicotine replacement therapy (NRT); however, only 10% of pregnant smokers use these services and as few as 3% stop smoking [14]. Effective approaches are limited. New interventions are needed to increase engagement with SSS, encourage uptake, support quit attempts and produce better outcomes [15].

### Stop smoking support

Stop smoking support is freely available to pregnant women throughout the UK. Models of support differ, however, depending on where the women live. In general, two main types of support are offered which can be described as ‘specialist’ (just for pregnant women) or ‘generic’ (for all smokers including pregnant women). Within this framework, support offered commonly includes: individual/group support provided by specially trained advisers who may be nurses or midwives; support provided in the hospital setting, women’s homes or another mutually acceptable venue; at least one face-to-face counselling session with follow-up support, often by telephone, to 12 weeks after a quit date is set; and advice on use of NRT utilising various models of prescribing (e.g. nurse/GP prescribing/pharmacy).

The National Institute of Health and Care Excellence (NICE)—PH26 Smoking: stopping in pregnancy and after childbirth [15]—published comprehensive guidance in 2010 regarding services that should be provided to pregnant smokers.

### Scientific premise for the trial

The rationale for incentives is that they can stimulate behaviour change through providing an immediate reward for changes in health behaviours (e.g. smoking cessation), which is likely to be more motivating to people than more distal rewards such as health improvements. For smokers who quit, the saving of not buying cigarettes provides continued ‘value’ long after incentives have stopped. Despite the unborn child having no choice regarding tobacco exposure, the ‘extra’ cost of incentives is a deterrent for policy-makers and planners and is linked to a societal moral judgement of ‘rewarding bad habits’ [16, 17]. However, public opinion towards financial incentives is mixed, and public acceptability increases with effectiveness [17, 18]. This study can justify the use of financial incentives by providing evidence to show whether this upstream preventive intervention [19, 20] can be cost-effective and much cheaper than trying to cure smoking-related conditions downstream.

### Evidence for use of financial incentives for stopping smoking during pregnancy

Published research using financial incentives for smoking cessation during pregnancy is limited to single-centre trials; however, as reported in two recent Cochrane reviews [21, 22], together they add up to a body of work indicating a beneficial effect that is likely to be cost-effective [23]. Combining data from nine trials of 2273 pregnant women, the 2019 review by Notley et al. [21] concluded that there is moderately certain evidence that women in the incentives groups were more likely to stop smoking than those in the control groups, both at the end of the pregnancy and after the birth of the baby—the RR at longest follow-up (up to 24 weeks post-partum) was 2.38 (95% CI 1.54 to 3.69; *N* = 2273; *I*^2^ = 41%), in favour of incentives. The 2017 review by Chamberlain et al. [22] reported that high-quality evidence suggests that incentive-based interventions are effective when compared with an alternative (non-contingent incentive) intervention (four studies; RR 2.36, 95% CI 1.36 to 4.09). Pooled effects were not calculable, however, for comparisons with usual care or less intensive interventions (substantial heterogeneity, *I*^2^ = 93%).

This body of research is still not sufficient to overcome concerns put forward by policy-makers regarding financial incentive payments [17] or to fully answer the first research question put forward by the NICE [15]: ‘Within a UK context, are incentives an acceptable, effective and cost-effective way to help women who smoke to quit the habit when they are pregnant or after they have recently given birth? Compared with current services, do they attract more women who smoke, do they lead to more of them completing the stop-smoking programme and do more of them quit for good? What level and type of incentive works best and are there any unintended consequences?’

To start to address these research questions in a UK context, our previous large (*n* = 612) single-centre feasibility trial in Glasgow, UK [24] added financial incentives to usual SSS care and compared outcomes with usual care alone. Smokers routinely identified at first maternity care visit were individually randomised to receive either usual SSS support only or the same support with the offer of financial voucher incentives. The first three vouchers were contingent on engagement with SSS. The last voucher (£200) could be earned by stopping without SSS support. Twenty-three per cent quit with the offer of usual care plus incentives (up to £400) and 9% with the offer of usual care alone (*p* < 0.001). A novel embedded health economic evaluation indicated that the intervention was highly cost-effective [23].

### Need for a further trial

The context within which incentives are offered is important. Socio-demographic, geographic and organisation differences may affect future transferability of the intervention and the potential to implement a sustainable intervention in the long term [25, 26]. Adding incentives to a range of SSS models in different areas of the UK serving varied population groups needs to be tested before clear recommendations can be made.

In addition, further evidence is required to inform the cost-effectiveness debate of incentive-based schemes. The economic analysis from our feasibility trial [23] indicated relapse post-partum was the biggest area of uncertainty. Six months is the recommended period to measure long-term abstinence [27], as those abstinent at this time point tend to remain smoke-free in the long term [28].

A pivotal phase III multi-centre UK trial that includes cessation outcomes to 6 months after birth is therefore required to be able to recommend changes in policy and practice [15], and thus for SSS funders (such as the NHS or local government in the UK) to consider this approach to smoking cessation as part of mainstream services.

The proposed study will assess whether promising feasibility trial findings [24] can be transferred to other UK sites with different SSS configurations and population groups. If found to be effective and cost-effective in this multi-site trial, the simple novel ‘bolt-on’ nature of the intervention will make the trial results generalisable to a wide range of SSS and population groups, and will allow easier transfer of the intervention to other SSS within the UK and other parts of the world.

### Objectives

This RCT will examine, within a range of usual care pathways, the effectiveness and cost-effectiveness of financial voucher incentives when offered in addition to usual SSS support, to encourage women to attend SSS and set a quit date, to quit smoking and be abstinent towards the end of pregnancy and at 6 months after birth.

The primary objective is to determine whether the offer of financial voucher incentives in addition to usual SSS support leads to a doubling of the smoking cessation rate by the end of pregnancy.

Secondary objectives are as follows:

- To compare quit rates at 4 weeks post quit date and 6 months after birth between women offered incentives and those receiving usual SSS care only
- To assess, from an NHS perspective, whether financial incentives are cost-effective in terms of cost per quitter (at birth and 6 months post-partum) and per quality-adjusted life year gained
- To identify the effect of differences in SSS and demographic diversity of pregnant smokers on the effectiveness, cost-effectiveness and transferability of financial voucher incentives
- To explore the barriers and facilitators to trial recruitment, retention and implementation in different areas

### Trial design

This study is a pragmatic, 42-month, multi-centre, parallel-group, single-blinded, individually randomised controlled superiority trial with 1:1 allocation designed to assess whether the addition of financial incentives to usual SSS helps pregnant women to stop smoking. In addition, an economic evaluation from a UK NHS perspective will assess cost-effectiveness of offering financial incentives added to usual SSS; a mixed-methods theory-driven [29, 30] process evaluation will examine barriers and facilitators to trial enrolment and future implementation of incentives in a range of contexts; and data from the feasibility trial centre in Glasgow [24], a deprived inner city, will also be analysed in an a priori meta-analysis.

**Fig. 1:**
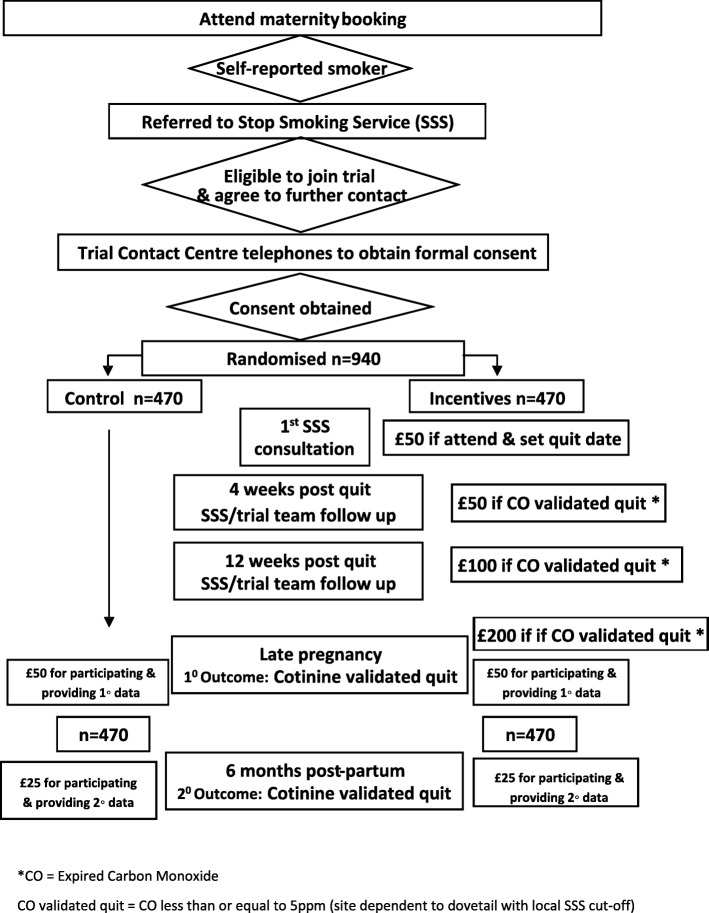
Overview of the trial design and flow of participants through the study. *CO = expired carbon monoxide. CO validated quit = CO less than or equal to 5 ppm (site dependent to dovetail with local SSS cut-off point)

An overview of the trial design is illustrated in Fig. 1.

## Methods

This protocol is reported according to the 2013 SPIRIT Guidelines [31].

### Study setting

Women will be recruited from SSS serving maternity hospitals in three of the four UK nations—Scotland, England and Northern Ireland. Participating sites include a deprived city, a deprived post-industrial suburban and rural area, a provincial city, two provincial towns, a deprived coastal city and a rural area. Each of these sites have different SSS configurations offering their own care pathway within the framework of the UK NICE guidance [15]. These include NHS/local authority-run services, generic/specialist pregnancy services, midwifery/SSS advisor-led services and opt-in/opt-out services, and represent most UK usual care pathways for smoking cessation in pregnancy. Each of the sites have between 1000 and 6000 deliveries per annum. The diversity of sites thus incorporates organisational differences and facilitates recruitment of a mix of women from different geographic and socio-economic backgrounds.

### Eligibility criteria

Eligible women for the trial are those who: are aged 16 years or over; are pregnant for less than 24 weeks gestation at maternity booking, or if not yet had their first antenatal appointment, less than 24 weeks gestation at time of consent; self-report as current smokers (at least one cigarette in the last week); live in the catchment area of the participating NHS site; and are able to understand and speak English in order to provide verbal telephone consent and follow-up smoking status.

### Intervention

Control group women will receive the offer of usual, local SSS support.

**Fig. 2:**
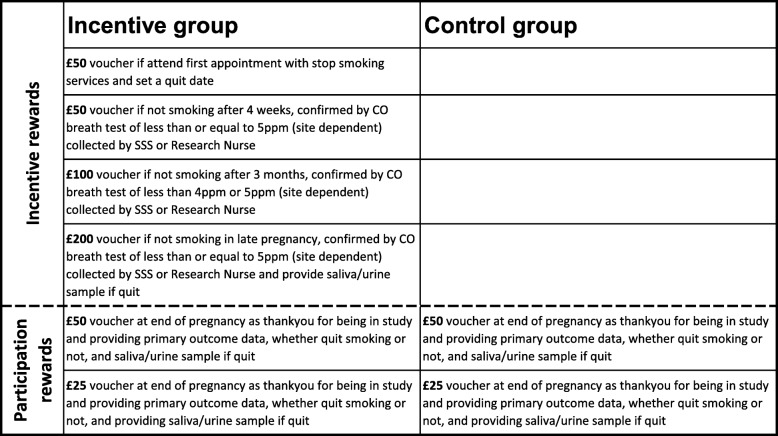
Incentive and participation rewards structure. CO carbon monoxide, SSS Stop Smoking Services

Intervention group women will receive the same offer of usual, local SSS support. In addition, they will be offered financial incentives up to £400 to engage with local SSS and set a quit date, and remain abstinent at each follow-up point throughout pregnancy. The incentives will be in the form of Love2Shop gift cards that can be redeemed in a wide variety of UK shops, none of which currently sell cigarettes. The incentive rewards structure is shown in Fig. 2.

#### Adherence with intervention

Women allocated to the intervention group will have the opportunity to receive shopping vouchers at four key time points in the trial, dependent on their smoking status. Consequently, adherence will be assessed by considering distribution and receipt of shopping vouchers, which will be confirmed by Royal Mail signature.

### Outcomes

#### Primary outcome

The primary outcome is cotinine/anabasine-verified abstinence from smoking for at least 8 weeks towards the end of pregnancy at 34–38 weeks gestation. The proportion of abstinent women will be compared between the intervention and control groups.

#### Secondary outcomes

Secondary outcomes include other key smoking cessation, child, health economic and process endpoints, and focus on the difference between the intervention and control groups regarding the following:

1. Proportion of women who engage with SSS (locally defined) and set a quit date
2. Proportion of women with biochemically validated (CO) self-reported abstinence from smoking for at least 14 days at 4 weeks after quit date
3. Proportion of women with cotinine/anabasine-verified self-reported point abstinence from smoking for at least 8 weeks at 6 months post-partum
4. Proportion of women with cotinine/anabasine-verified self-reported continuous abstinence from smoking from late pregnancy to 6 months post-partum
5. Mean difference in birth weight
6. Cost-effectiveness: incremental cost per late pregnancy quitter and cost per quality-adjusted life year (QALY) gained over the trial time horizon and lifetime
7. Process evaluation: barriers and facilitators to trial recruitment and future implementation of incentives in practice

Data for the primary outcome and secondary outcomes 1, 2 and 5 will be combined with data from the feasibility trial in a meta-analysis, as described in ‘Statistical methods’.

### Sample size and recruitment

The sample size for this phase III trial is 940 pregnant smokers. This was calculated on the basis of the primary outcome. A total of 940 participants (470 in each group) will detect a clinically significant doubling of the cotinine-validated quit rate from 7% with usual care alone to at least 14% with usual care plus the offer of financial voucher incentives, with 90% power at the 5% significance level allowing 15% loss to follow-up.

Eligible pregnant smokers will be enrolled over a 26-month period from February 2018 to March 2020. Recruiting for 18 months would have allowed all participants to be followed up to the secondary outcome point 6 months after birth. Recruiting for 26 months (including a 3-month funded extension from CRUK—see Funding for confirmation) has allowed an additional 8 months with recruitment slower than expected whilst allowing the first 75% of participants recruited to be followed up to the secondary outcome point 6 months after birth. This compromised 6-month post-partum follow-up was agreed with the funders, the ethics committee and the sponsor prior to the study start in September 2017.

### Allocation and blinding

Enrolment and randomisation will be performed over the telephone by GCP-trained call centre staff at the Database Management Company (Trial Contact Centre (TCC)) once the women’s contact details and eligibility data have been submitted to the secure online trial database by research staff. All calls will be audio-recorded and information obtained during the call entered directly into the database. After obtaining informed consent and baseline data, TCC staff will then press the on-screen button to randomise women and inform them of their group allocation. TCC staff will not be able to influence or predict the random allocation which is integrated into the database.

The random allocation sequence will be generated by York Trials Unit. Women will be allocated 1:1 to either the intervention or the control group using randomly varying permuted block sizes with no stratification factors. In addition, a random date between 34 and 38 weeks gestation for each pregnancy will be generated as the date for primary outcome data collection. This date will be concealed from both the TCC staff and the women.

It will not be possible to blind women or research nurses to group allocation. The TCC staff responsible for ascertaining the primary outcome measure of self-reported smoking status in late pregnancy (corroborated by saliva cotinine measurement collected by a research nurse) will, however, be blind to allocation. Women will be asked not to disclose their group status during the follow-up telephone call with the TCC. The statistician conducting analyses will have no contact with women but will not be blind to treatment allocation.

### Participant timeline and data collection

**Fig. 3:**
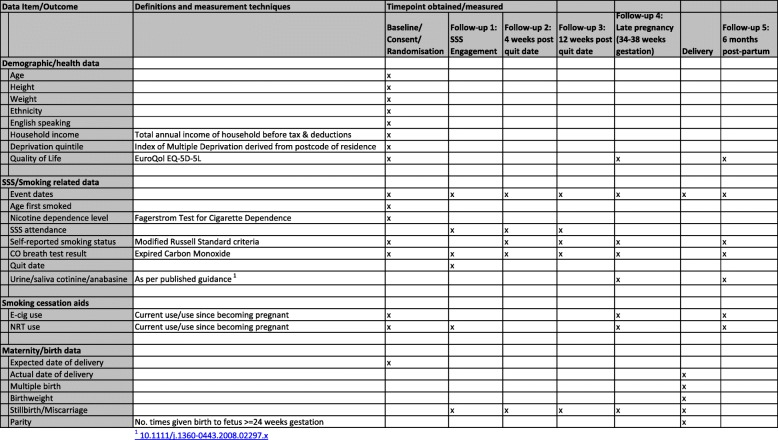
Schedule of assessment and data collection for trial outcomes. ^1^CO carbon monoxide, E-cig e-cigarette, NRT nicotine replacement therapy, SSS Stop Smoking Services

The trial consists of an intervention phase between 6 and 38 weeks gestation with five assessment points and follow-up to 6 months post-partum. The total study period for each participant will be 42–62 weeks dependent on gestation at enrolment and timing of primary outcome assessment in late pregnancy (randomised between 34 and 38 weeks gestation). See Fig. 1 for an overview of the study design and measurement time points, and Fig. 3 for the schedule of assessment and data collection.

#### Identification and recruitment of participants

Information about the trial will be displayed in appropriate clinical areas. Following antenatal assessment, pregnant smokers referred to SSS will be assessed for eligibility by local SSS or trial research staff. During their first routine contact with SSS, eligible women will be given information about the trial. Those who are interested in taking part will be asked to give verbal permission for further trial contact and for personal details to be passed to the TCC to allow informed telephone consent. The SSS will then continue with usual care and follow-up. If necessary (depending on trial information provided during first routine SSS contact), local research staff will telephone women to further discuss the trial prior to the scheduled consent call. On receipt of personal details at the TCC, a letter and a participant information sheet (PIS) will be automatically sent to women by post. Three days after this, an alert will be sent to those women who agreed to text message contact to remind them of the 0800 number that the TCC will call them from to discuss the study and obtain consent.

#### Consent and randomisation

At least 5 days after posting the PIS, the TCC will contact women to undergo formal consent procedures. Telephone contact will be attempted on a minimum of three and a maximum of eight occasions, where possible, at the time slot preferred by the client—weekday am/pm/evening or weekend am/pm—after which no further attempts at enrolment will be made. At the start of the consent call, call handlers will confirm eligibility and receipt of the PIS. Those who report not having received the PIS will be given the option of a verbal summary or to have another copy sent to them and called back in a few days. On proceeding, 15 consent questions will follow, six of which women must answer and accept to participate in the trial. These include consenting to access to hospital records where appropriate to the trial. One of the remaining nine questions will ask women to consent to trial staff accessing ‘left-over blood’ from routine samples collected in late pregnancy. The consent form is shown as backmatter.

After giving informed consent, women will be asked baseline questions measuring the level of addiction to cigarettes (Fagerström Test for Cigarette Dependence [32]), partner smoking, quality of life (EQ-5D-5L [33]), household income and use of nicotine alternatives (e.g. NRT or electronic cigarettes). At the end of the telephone call, women will be randomised and informed of their group allocation and an automated study pack (copy of consent form showing group allocation and PIS) will be sent to women in the post. Audio-recordings of the consent process will be stored in accordance with Good Clinical Practice guidelines.

#### Follow-up 1: SSS engagement

After women have consented and been informed of their group allocation, trial research staff will contact their local SSS to ascertain whether women attended a first appointment with an SSS advisor and set a quit date. This information will be entered into the trial database for both control group and intervention group women. A £50 voucher will automatically be dispatched to intervention group women who attended and set a quit date.

#### Follow-up 2: 4 weeks post quit date

For those women who engaged with the SSS and set a quit date, trial research staff will contact their local SSS 4 weeks after this quit date to obtain their smoking status in the last 2 weeks and CO breath test result as recorded by the SSS. Where a breath test result is not available from the SSS trial, research nurses will collect this directly from the woman for the incentives group to initiate incentive payments. CO breath test results will be collected for the control group only where these are available from the SSS in line with national SSS guidelines. This information will be entered onto the trial database. If the CO result is at or below the accepted level for a non-smoker at the site, a £50 voucher will automatically be dispatched to women in the incentives group.

#### Follow-up 3: 12 weeks post quit date

For those women in the intervention group who were confirmed quit at 4 weeks, trial research staff will contact their local SSS 8 weeks later to obtain their smoking status and CO breath test result as recorded by the SSS. Where this is not available from the SSS trial, research nurses will collect this directly from the woman. This information will be entered into the trial database. If the CO result is at or below the accepted level for a non-smoker at the site, a £100 voucher will automatically be dispatched

#### Follow-up 4: late pregnancy (34–38 weeks gestation)

All women will be followed up at the primary outcome stage in late pregnancy. Follow-up telephone contact will be attempted by the TCC at a random date between 34 and 38 weeks gestation allocated at the time of initial randomisation. Trial research nurses will review the women’s notes 1 week prior to the telephone contact to check the health status of mother and baby and to alert TCC staff to any adverse events (e.g. miscarriage or stillbirth) that may require particular sensitivity when conducting follow-up. TCC staff will be blind to group allocation.

Three attempts will be made by the TCC to contact women. If no contact is established women will be followed up by local research staff by telephone, text and letter. On successful contact, women will be asked ‘Have you smoked in the last 8 weeks?’ If yes, ‘Have you smoked more than 5 cigarettes in that time?’ EQ-5D-5L data [33] and current NRT/electronic cigarette use will also be collected at this time point.

Self-report of not smoking will be corroborated by cotinine estimation on saliva or urine (when saliva collection cannot be tolerated). Where women are also using NRT or electronic cigarettes, anabasine assay on urine will replace cotinine. Cotinine and anabasine will be assayed by ABS Laboratories Limited. To minimise the potential for women to ‘game’ the primary outcome, incentive payments will be dependent on the CO result, which is an immediate measure, and not on the cotinine or anabasine level.

An important aspect of the primary outcome for this phase III trial is the proportion of women successfully followed up in both the intervention and control groups. To minimise loss to follow-up, particularly among controls, women in both groups will receive Love2Shop vouchers of £50 and £25 for providing data and saliva/urine samples where applicable at the primary (late pregnancy) and secondary (6 months post-partum) outcome time points, respectively (Fig. 2). Acceptable levels are around 90% of participants successfully followed up in each group.

To assess whether women lost to trial follow-up are still smoking towards the end of pregnancy and whether the primary outcome has been ‘gamed’ (saliva cotinine below the cut-off point but still smoking in late pregnancy), residual blood from routine late pregnancy samples, where available, will be tested.

#### Follow-up 5: 6 months post-partum

Similar to the late pregnancy follow-up, all women will be contacted at 6 months after their expected delivery date to ascertain their smoking status and collect a saliva/urine sample for those women who self-report as quit. Quit status 6 months after birth will be ascertained by two sets of questions:

1. ‘Have you smoked in the last 8 weeks?’ If yes, ‘Have you smoked more than 5 cigarettes in that time?’
2. ‘Have you smoked since your baby was born?’ If yes, ‘Have you smoked more than 5 cigarettes in total since your baby was born?’

Follow-up procedures (i.e. number of contact attempts, data collection and saliva/urine sample collection and assay) will be the same as those described for the late pregnancy follow-up. Biological samples of saliva and urine will not be available for use by other researchers.

#### Birth-related data collection

After the expected date of delivery, research nurses at each site will collect and input into the trial database data regarding parity, baby’s birth date and weight.

### Data management

The data management process will be run by York Trials Unit. The protocol was built on the platform from the phase II trial [34]. This has been improved and updated by York Trials Unit in conjunction with the central trial team (DMT, LS and MM) and has been used for submissions for regulatory approval.

The database is a modified version of that used in CPIT II. York Trials Unit, central trial management in Glasgow and research staff at one of the recruiting sites have contributed to the design of the modified version.

Data entry will be completed by trained research staff at local sites.

### Statistical methods

Statistical analysis will be conducted by York Trials Unit (AM and AK). All analyses will be carried out using the intention-to-treat principle unless stated otherwise. Treatment effect estimates will be presented along with the corresponding 95% confidence interval, and statistical tests will be two-sided at the 5% level, unless otherwise stated.

#### Primary outcome analysis

Primary outcome analysis will be by intention to treat as the intervention is the offer of a financial incentive to engage with SSS and quit smoking. Logistic regression will adjust for maternal age, years of smoking, deprivation score, level of smoking and site.

#### Secondary outcome analysis

Engagement with SSS and self-reported smoking status at 4 weeks will both be analysed using a logistic regression model adjusting for the same covariates as the primary analysis. Continuous and point abstinence (i.e. regardless of whether participants were abstinent in late pregnancy) outcomes obtained at 6 months post-partum [28] will be calculated using logistic regression also adjusting for the same covariates as the primary outcome analysis. For each of the following covariates, tests for interaction with the treatment group will be performed: age of mother, years of smoking, deprivation score and level of smoking. Effects on the length of neonatal unit stays will be examined.

Birth weight will be analysed using a linear regression model adjusting for key prognostic variables including age, site, height and weight of mother at early pregnancy. The intention-to-treat estimate will be severely diluted due to low smoking cessation rates and the ‘per protocol’ analysis will be biased by confounding. Consequently, we will also utilise an instrumental variable approach—complier average causal effect analysis—which will estimate the true impact of incentive-induced smoking cessation on birth weight [35].

Differences by subgroup (e.g. site, deprivation, age group) will be explored and reported as per the CHAMP guidelines [36].

A meta-analysis including data collected in the feasibility study in Glasgow on 612 participants [24] will be undertaken.

#### Missing data

Where there are missing data for the primary outcome (i.e. smoking status) it will be assumed that women are continuing to smoke. This assumption will be examined by testing residual blood samples (taken for other reasons in late pregnancy) for cotinine, as in the feasibility trial [24]. This assumption will also apply to the 6-month post-partum secondary outcome of smoking status. Other secondary outcomes (e.g. birth weight) are collected routinely and will have few missing data. Long-term outcome data collection will be planned from participants and offspring to inform additional follow-up studies.

### Economic and process evaluations

An economic evaluation to assess cost-effectiveness of offering financial incentives in addition to routine SSS will be undertaken from an NHS perspective. Details are the subject of an additional protocol paper to be published separately.

A process evaluation using a mixed-methods case-study approach will explore recruitment and assess ‘intervention context fit’. This is essential to understand both how the trial functions within different SSS and how applicable and generalisable the findings may be in terms of future implementation. Full details of the process evaluation design and methods are reported in an Additional file 1.

### Data monitoring

Data monitoring will be coordinated by York Trials Unit and includes some self-monitoring at sites (see backmatter).

Serious adverse events (SAEs) that are related to the intervention will be documented. It is not anticipated that the provision of shopping vouchers to women will be associated with any related SAEs. SAEs in the feasibility study were primarily due to miscarriages that were not related to the intervention. For this reason, a separate Data Monitoring Committee will not be assembled.

Stopping the trial for reasons not related to safety such as ‘futility because the required sample size cannot be reached’ will be decided by the Trial Steering Committee.

Data cleaning will be conducted by York Trials Unit (AM) and the central trial management team in Glasgow (LS).

## Discussion

At present, only 10–20% of pregnant smokers take up the offer of free SSS and only 3–8% quit during pregnancy with usual care that includes counselling and NRT. Modest incentive payments to engage with SSS and/or to quit smoking may provide a substantial benefit by decreasing pregnancy and first-infant year health care costs. If women stay smoke-free, long-term health care costs will be substantially reduced. The results of this phase III multi-centre trial will examine the costs and benefits of providing financial incentive payments for smoking cessation during pregnancy across the UK. This evidence will provide information required for NICE to consider recommending financial voucher incentive payments to support pregnant smokers to quit across the UK at the scheduled 2021 guideline PH26 [15] update Additional file 2.

## Trial status

Recruitment opened in February 2018 and will be complete by the end of March 2020. On 17 December 2019, 837 of 940 participants were enrolled into the trial.

Current protocol V3.1, 27 September 2018.

## Supplementary information

**Additional file 1.** Mixed methods process evaluation study protocol [37–40].

**Additional file 2.** SPIRIT 2013 Checklist: Recommended items to address in a clinical trial protocol and related documents.

## Abbreviations

CI: Confidence interval
CPIT: Cessation in Pregnancy Incentives Trial
GG&C: Greater Glasgow & Clyde
NHS: National Health Service
NICE: National Institute for Health and Clinical Excellence
NRT: Nicotine replacement therapy
QALY: Quality-adjusted life year
