# Supplementary material for: The smoking cessation in pregnancy incentives trial (CPIT): study protocol for a phase III randomised controlled trial
Source: Trials. 2020 Feb 14;21:183. doi: 10.1186/s13063-019-4042-8 (PMC7023794; doi:10.1186/s13063-019-4042-8)
Supplement: Supplementary file 1 — Additional file 1. Mixed methods process evaluation study protocol [37–40]. [file 13063_2019_4042_MOESM1_ESM.docx]

**Mixed methods process evaluation study protocol**

*Study conduct and design*

The process evaluation will be conducted by the University of Stirling, led by experienced qualitative researchers JM and IU, under the direction and supervision of FH (qualitative expertise) and PH (clinical and mixed methods expertise). This work will expand on qualitative research already undertaken with trial participants and professionals in relation to CPIT II, the pilot incentives trial undertaken in Glasgow [24], and research with parents and health professionals on the acceptability and mechanisms of action of financial incentives [37].

The mixed methods process evaluation applies a longitudinal case study design [29, 30] informed by the realist evaluation approach [38]. Each case is defined as a trial site(a SSS serving a maternity hospital) and data collection will be conducted in two stages. Stage 1 (service and local context assessment) will consist of field notes from structured observations and unstructured interviews with local site maternity and smoking cessation professionals during trial set-up and recruitment phases of the trial to establish the characteristics of each cessation service and usual care for pregnant smokers, as well as the context for delivering the trial. Trial participants will not be directly involved in Stage 1.

Stage 2 (trial impact) will consist of observations of regular trial management and local site meetings, where held, and semi-structured audio-recorded interviews with 4 to 8 key staff involved with conducting the trial, and CPIT participants (n=up to 6 in each site). Routine quantitative data from smoking cessation services in each site will also be obtained in relation to rates of referral, retention and attrition to examine these in the 12 months (at least) prior to, and the 12 months following the opening of the trial locally. Additionally, a sample of enrolment calls with eligible smokers will be obtained from the Contact Centre for analysis within the process evaluation.

A random sample of calls made by selected call agents to intervention and control participants from each site will be analysed for fidelity to trial processes, barriers and enablers to recruitment..

*Sampling and recruitment for interviews*

Purposive sampling for interviews with professionals will target two to four smoking cessation service staff, one to two service managers and two to four midwives at each site, as well as one to two call agents from the Contact Centre. Recruitment will be facilitated by site leads and local service managers.

At recruitment and after primary outcome data collection the contact centre will request consent from trial participants to be contacted by a researcher to discuss their experience of CPIT. Up to six interviews are planned with participants from each site, and these will include those in both intervention and control groups. A sampling frame will be used to ensure diversity for maternal age, ethnicity and socio-economic background and address under-represented groups in the qualitative data from the CPIT II trial. An iterative process informed by concurrent qualitative analysis and including live trial eligibility, recruitment and attrition data will also inform the sampling strategy and topic guides for data collection. Efforts will be made to capture the perspectives of eligible smokers who agree to have their contact details passed to the Contact Centre but who do not respond to call attempts to enrol in the trial.

All women (trial participants and non-participants) who take part in a telephone interview will be offered a £25 Love2Shop voucher as a thank-you for their participation.

For a period during trial recruitment an extra consent question will be ‘turned on’ during the consent call asking clients also to consent to being contacted by trial staff for the purposes of one-to-one interviews to fulfill qualitative process evaluation aspects of the trial.

*Data Collection*

Interviews will be facilitated by JM or IU and will follow a semi-structured topic guide, informed by previous qualitative research on incentives for smoking cessation; issues arising from the trial around recruitment, retention and engagement; and from internal trial team discussions. Prior to every interview, we will ensure that interviewees provide informed consent, based on the provision of study information in advance and the opportunity to ask questions. Interview recordings will be transcribed verbatim with the resulting transcripts anonymised, only to be shared with members of the research team involved in the process evaluation analysis (JM, IU, FH and PH). JM and IU will only share data with the team where confidentiality can be maintained. It is acknowledged that clinical staff in particular may well be identifiable therefore care will be taken with reporting to ensure that all quotes used are not attributable to individuals.

Interviews with women who consent to take part in the trial will take place over the phone and last between 30-45 minutes depending on what interviewees have to say and their availability. Interviews will explore experiences and views of taking part in the trial including barriers and facilitators, as well as views on recent support for smoking cessation. The option of a shorter interview of ten to fifteen minutes will be offered to women who decline to take part in the trial. These interviews will consider views on being approached to take part in the study and what changes, if any, could be made to make it easier for women to participate in the trial.

Interviews with relevant professionals will be conducted face to face or over the phone and, as with participants, last between 30 and 45 minutes or according to their availability. Professional interviews will explore barriers, facilitators and contexts of conducting the trial (local capacity, organisational structures and any changes to these); and fidelity to trial processes (recruitment to the trial, information given to patients, training issues).

Where agreeable to smoking cessation services in sites and eligible smokers, researchers will also observe (and with consent possibly audio-record) phone calls made by smoking cessation advisers to offer cessation support and introduce the trial.

Interview and observational data collected from sites will be supplemented by field notes of regular meetings involving trial staff, including managers and local research nurses, in order to reflect on recruitment data, and barriers and facilitators to trial implementation. Lessons learned regarding successful referral and recruitment strategies will be shared across sites in order to facilitate best practice in trial recruitment. Any changes or improvements made to these processes will be included in data analysis. Enrolment calls to intervention and control group participants from each site, sampled from selected call agents will be analysed to understand any variation in recruitment rates between sites.

*Data Analysis*

Analysis of the process evaluation data will explore the process of change longitudinally across the case study sites, including both within case and cross case comparison. Within case analysis will detail and explore site characteristics such as service configurations, geography and population demographics that might impact on recruitment and trial delivery, and provide a basis for developing context, mechanism and outcome (CMO) configurations. This will assist in identifying any active mechanisms and facilitating (or impeding) contexts that may impact on outcomes in each site. Cross case comparison will allow for identification of higher level themes and learning from across the sites including barriers and facilitators to trial delivery within health services and the implementation of financial incentives within smoking cessation support services.

The framework method [39] will structure the process evaluation analysis. Transcribed interviews, observations and quantitative data for expected and actual recruitment targets will be entered into QSR NVivo (v12) software to facilitate the indexing stage and during the ‘charting’ phase we will construct matrices to compare and contrast data both within and across trial sites. Independent reading of up to four transcripts by the process evaluation team (JM, IU, PH and FH) will inform the drafting of an initial coding index. Two researchers will code at least two transcripts and two sets of observation notes each to test the index and will meet to discuss and agree any themes arising that are not included. A finalised coding index will then be agreed and applied to all transcripts and observation notes and used to populate a framework matrix for further analysis and interpretation within and across cases.

The realist evaluation approach will seek to identify the active mechanisms that are likely to promote successful future implementation of the intervention, if it is shown to be cost-effective and link these to relevant contextual factors. This will provide important insights for policy and decision makers.

Quantitative and qualitative results will be reconciled as part of the process evaluation, according to a well recognized triangulation protocol, examining themes for dissonance and convergence [40].
